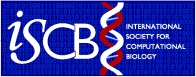# ISMB 2006

**DOI:** 10.1371/journal.pcbi.0020011

**Published:** 2006-02-24

**Authors:** Goran Neshich, Philip E Bourne, Søren Brunak


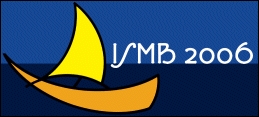


The International Conference on Intelligent Systems for Molecular Biology (ISMB) is the premier meeting of the International Society for Computational Biology (ISCB), drawing up to 2,000 attendees annually (Box 1). This year is of particular significance as the meeting will be held in Fortaleza, Brazil, August 6–10 (http://www.iscb.org/ismb2006), as opposed to sites in Europe and the United States. The location reflects the ISCB's role in promoting computational biology worldwide and its recognition of Brazil's emergent role in the fast-growing field of computational biology. ISMB 2006 will be preceded by the 20th anniversary of SwissProt (http://www.swissprot20.org), allowing for an extended period of high-quality science and the opportunity to see a unique part of the world.

ISMB 2006 will be notable by having keynote lectures from three Nobel Laureates (Rich Roberts, Kurt Wuthrich, and Robert Huber), as well as Sir Tom Blundell, Charles DeLisi, Elena Conti, Mathieu Blanchette and Michael Waterman. While still including the core scientific areas found at previous ISMB meetings (e.g., evolution and phylogeny, comparative genomics), the program will be extended to include sessions in keeping with the scope of *PLoS Computational Biology—*e.g., a session on the computational impact on human health, a distinguished panel discussion on “New Frontiers in Computational Biology,” and sessions on systems biology. A special session on “Bioinformatics in Latin America” will reveal some outstanding contributions to the variety of computational biology–related fields, most notably, the applications in plant- and human-related diseases. Papers accepted to the conference will be published as an open-access issue of the journal *Bioinformatics*. If previous meetings are indicative, over 400 papers and 600 posters could be submitted. To accommodate late-breaking research, selected posters will be presented orally in a PLoS sponsored session.

Area chairs are still being secured, but to date the following distinguished scientists have agreed to manage the review process in at least the following broad topic areas: (1) Manolo Gouy and Tandy Warnow, evolution and phylogeny; (2) researchers soon to be determined, human health; (3) Minoru Kanehisa and Rolf Apweiler, databases and data integration; (4) Michael Claverie and Eugene Koonin, comparative genomics; (5) Eugene Shakhnovich and Diana Murray, molecular and supramolecular dynamics; (6) Anand Kumar and Robert Stevens, ontologies; (7) Joel Bader, systems biology; Zhaolei Zhang, proteomics; Nick Grishin and Søren Brunak, sequence analysis; Janet Thornton and Robert Russell, structural bioinformatics; Alfonso Valencia and Andrey Rzhetsky, text mining and information extraction; Hanna Margalit and Michael Zhang, transcriptomics.

Finally, there will be software demonstrations, tutorials (some of which we hope to publish in *PLoS Computational Biology*), meetings of special interest groups, and more. With the promise of great science and Brazilian hospitality waiting, we look forward to seeing you in Brazil.